# An Experimental Comparison between Deep Learning and Classical Machine Learning Approaches for Writer Identification in Medieval Documents

**DOI:** 10.3390/jimaging6090089

**Published:** 2020-09-04

**Authors:** Nicole Dalia Cilia, Claudio De Stefano, Francesco Fontanella, Claudio Marrocco, Mario Molinara, Alessandra Scotto di Freca

**Affiliations:** Department of Electrical and Information Engineering “Maurizio Scarano”, University of Cassino and Southern Lazio, 03043 Cassino (FR), Italy; nicoledalia.cilia@unicas.it (N.D.C.); destefano@unicas.it (C.D.S.); c.marrocco@unicas.it (C.M.); m.molinara@unicas.it (M.M.); a.scotto@unicas.it (A.S.d.F.)

**Keywords:** paleography, writer identification, deep learning, classification systems, feature extraction

## Abstract

In the framework of palaeography, the availability of both effective image analysis algorithms, and high-quality digital images has favored the development of new applications for the study of ancient manuscripts and has provided new tools for decision-making support systems. The quality of the results provided by such applications, however, is strongly influenced by the selection of effective features, which should be able to capture the distinctive aspects to which the paleography expert is interested in. This process is very difficult to generalize due to the enormous variability in the type of ancient documents, produced in different historical periods with different languages and styles. The effect is that it is very difficult to define standard techniques that are general enough to be effectively used in any case, and this is the reason why ad-hoc systems, generally designed according to paleographers’ suggestions, have been designed for the analysis of ancient manuscripts. In recent years, there has been a growing scientific interest in the use of techniques based on deep learning (DL) for the automatic processing of ancient documents. This interest is not only due to their capability of designing high-performance pattern recognition systems, but also to their ability of automatically extracting features from raw data, without using any a priori knowledge. Moving from these considerations, the aim of this study is to verify if DL-based approaches may actually represent a general methodology for automatically designing machine learning systems for palaeography applications. To this purpose, we compared the performance of a DL-based approach with that of a “classical” machine learning one, in a particularly unfavorable case for DL, namely that of highly standardized schools. The rationale of this choice is to compare the obtainable results even when context information is present and discriminating: this information is ignored by DL approaches, while it is used by machine learning methods, making the comparison more significant. The experimental results refer to the use of a large sets of digital images extracted from an entire 12th-century Bibles, the “Avila Bible”. This manuscript, produced by several scribes who worked in different periods and in different places, represents a severe test bed to evaluate the efficiency of scribe identification systems.

## 1. Introduction

Palaeography aims at studying mediaeval handwritings, and its objectives include, among the others, to ascertain when a manuscript was written, how many people wrote it and how they shared the work among them [1]. In this context, “traditional” studies are performed by human experts who are able to identify the characteristics of a manuscript by using traditional palaeographical tools, e.g., protractors and set squares, to measure letter heights and widths, distances between characters and angles of inclination. In the last years, there has been a growing interest by paleographers in the use of computer-based techniques, which allow them to characterize medieval manuscripts in a more effective way [2,3]. This interest gave rise to a new research field generally known as digital palaeography, that has been also favored by the ever-increasing availability of tools and methodologies for the acquisition of high-quality digital images. Digital technologies can be used either to perform traditional measurements more rapidly and systematically than in the past or to apply recently developed techniques based on artificial intelligence. Such techniques can be used to develop decision support systems to help paleographers in their tasks: in this case, the identification of effective features is of a paramount importance to obtain satisfactory results.

As concerns the feature extraction process, pattern recognition experts typically use two different approaches, namely “local” and “global”. The first one is focused on the characterization of the handwritten trace, while the second one extracts information related to the handwritten page as a whole, typically using texture features and/or layout analysis. More recently, there has been a growing scientific interest in the use of techniques based on deep learning (DL) for the automatic processing of ancient documents. These techniques, in fact, allow the system to automatically extract features from raw data, without using any a priori knowledge. This aspect is very important in the digital palaeography research field, which is characterized by an enormous variability in the type of ancient documents to be managed, due to the different historical periods, language and styles in which they were produced. The effect is that it is very difficult to define standard techniques that are general enough to be effectively used in any case, and typically requires the development of ad-hoc systems for each ancient manuscript, generally designed according to paleographers’ suggestions. Further details on these approaches will be provided in the next section.

In previous studies on these topics, we followed two distinct lines of research, with the aim of developing an end-to-end system that receives in input the images of pages of a medieval Latin books and it is able to assign each of them to one of the scribes who contributed to the writing process of such a book. In the first research line, we devised a set of features to characterize highly standardized handwriting and book typologies according to the suggestions of the paleographers. These features contain information related to the page organization, and to the scribe ability in exploiting the best the available space [4,5]. In the second research line, we investigated the use of DL techniques based on transfer learning, to develop a system for scribe identification.

Moving from the above considerations, the aim of this study is to verify if DL based approaches may actually represent a general methodology for automatically designing machine learning systems for palaeography applications. To this purpose, we compared the performance of a DL based approach with that of a “classical” machine learning one, in a particularly unfavorable case, namely that of highly standardized schools. In this case, indeed, the selection of some basic features, directly derived from page layout analysis, can be very helpful for automatically distinguishing the different scribes who produced the text. Following palaeographers’ suggestions, these features can be easily extracted by using standard image processing algorithms and allow the development of effective machine learning systems for scribe identification. On the contrary, DL systems extract the information useful to distinguish the different scribes using only the images containing fragments of text belonging to the ancient manuscript.

In other words, the rationale of this contribution is to verify whether DL-based approaches are able to extract discriminant features without exploiting any contextual information, whose presence and relevance can only be determined by palaeography experts: this result would confirm the generality of DL-based approaches and their applicability on any type of ancient documents, without requiring any a priori knowledge. In this study we also analyzed the error-reject curve for both DL systems and layout features based systems, applying the same reliability measure to both of them. To this aim, we first identify the rows in each page of an ancient document and assign each of them (or groups of them) to a single scribe, by using both systems. Then, we use the same rule to combine the obtained responses, associating the whole page to a single scribe (see Section 5). The combining rule provides also the overall classification reliability, thus allowing the implementation of a rejection rule. It is worth noticing that the possibility of introducing a rejection option is very important in palaeography applications, because it would allow palaeographers to verify only those pages of an ancient document that have been attributed to a specific scribe with a reliability deemed not sufficient.

The experimental results refer to the use of a large sets of digital images extracted from an entire 12th-century Bibles, the “Avila Bible”. This manuscript had a very complex history: it was written in Italy by at least nine scribes within the third decade of the 12th century. Subsequently, it was brought to Spain where other scribes worked on both text and decorations. Finally, in the 15th century, other additions were made by another scribe. The analysis of this manuscript is therefore very complex, due to the presence of several scribes who have worked in different periods and in different places, and represents a very significant test to evaluate the efficiency of scribe identification systems.

## 2. Related Work

As anticipated in the Introduction, in Digital Palaeography the feature extraction problem has been faced by following two different approaches, namely “local” and “global”. According to the first one, individual letters, signs, and their composing strokes are analyzed in order to derive relevant information. Examples of local approaches proposed in the literature, covering a wide spectrum of strategies, include the estimation of character curvature [6], the extraction of features based on the statistics of ink strokes [7], or the metrics to characterize the behavior of the writers. Moreover, because of the difficulties due to a correct character segmentation in degraded documents, methods based on word spotting have been also used [8,9].

As regards the global approaches, they typically extract information related to the handwritten page as a whole, by using texture features and/or layout analysis. In [10] the authors underline the limit of local approaches, and suggest the use of allograph and texture features. Following this line of thought, several approaches have been proposed for both writer identification [11,12] and document dating [7,13]. Moreover, in [11], the authors adopt texture-based features for the identification of the writers of the dead sea scrolls.

In recent years there has been an increasing number of studies based on DL techniques for the automatic processing of ancient documents. As noticed in the introduction, the interest in the use of such techniques is not only due to their capability of designing high-performance pattern recognition systems in a wide range of application fields [14,15,16,17], but also to their ability to automatically extract features from raw data, allowing the implementation of incremental learning algorithms [18]. In [19] the authors present a novel DL-based approach for recognizing an input image of multiple text lines from Japanese historical documents without explicit line segmentation. Moreover, Nguyen et al. [20] propose a segmentation-based method for digitizing nom documents using deep convolution neural networks, whereas Ziran et al. [21] introduce a novel technique for transcript alignment in early printed books by using deep models in combination with dynamic programming algorithms.

## 3. Layout Features for Writer Identification

**Figure 1:**
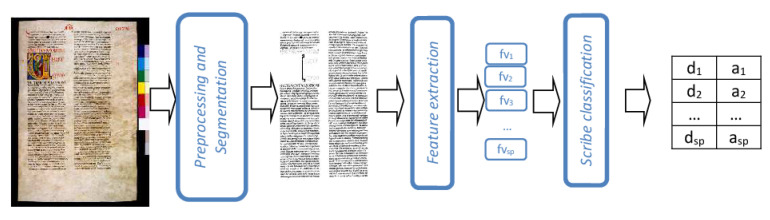
The architecture of the layout features (LF) System.

The architecture of the writer identification system based on layout features (LF systeming) is shown in Figure 1. The most important and original part of such system is the feature extraction step that has been developed in collaboration with experts in palaeography [4,5]. Before moving on to the description of the layout features, we briefly describe the preliminary steps of the LF system, namely pre-processing and segmentation. In the pre-processing step, noisy pixels, i.e., those corresponding to stains or holes on the page, or pixels included in the frame of the image, are detected and then removed. Red out-scaling capital letters, that might be all written by a single scribe, specialized for this task, are also removed. Finally, the RGB image is transformed into a binary black and white one. In the segmentation step, columns and rows in each page are detected by computing pixel projection histograms on the horizontal and vertical axis, respectively.

The feature extraction step generates a feature vector fv for each row (or group of rows as explained in the following) composed of three main sets of features. The first set includes the upper and the lower margin of the page and the inter-column distance. Such features, not very distinctive for an individual copyist, can be very useful to highlight chronological and/or typological differences.

The second set of features measure column properties: the number of rows in the column and the column exploitation coefficient [22]. The latter is a measure of how much the column is filled with ink, and it is computed as the ratio of the number of black pixels on the total number of pixels in a column. Both features vary according to different factors, among which the expertise of the writer. In the case of very standardized handwritings, such as the “Carolingian minuscule” of the “Avila Bible”, the regularity in the values assumed by such features may be considered as a measure of the skill of the writer and may be very helpful for scribe distinction.

The third set includes the following features: weight, modular ratio, interline spacing, modular ratio/interline spacing ratio and peaks. The weight is a measure of how much a row is filled with ink and it is computed as the ratio of the number of black pixels on the total number of pixels in a row. The modular ratio estimates the dimension of handwriting characters, and it is a typical palaeographic feature. According to our definition, this feature is computed for each row measuring the height of the “centre zone” of the words in that row. Once the centre zone has been estimated, the interline spacing is the distance in pixels between two rows. Modular ratio, interline spacing and modular ratio/interline spacing ratio characterize the way of writing of a single scribe and they may also hint to geographical and/or chronological distinctions. Highly discriminating features, such as the inter-character space and the number of characters in a row, imply the very difficult task of extracting the single characters contained in each word, which is far from being solved in the general case. Therefore, we chose to estimate the number of characters in a row by counting the number of peaks in the horizontal projection histogram of that row.

In order to reduce the variability in the above measures, due to due to specific inaccuracies that can occur locally in the writing process, we chose to average the feature values on *M* consecutive rows, rather than on a single row: the optimal value for the parameter *M* has has been experimentally determined. Summarizing, denoting with $n_{W}$ the number of different copyists to be distinguished, with $s_{P}$ the number of patterns selected in the page *P*, each composed of *M* consecutive rows, the feature extraction step generates $s_{P}$ feature vectors, each containing 10 real values.

Finally, the scribe identification step performs the recognition task, assigning each pattern of the input page *P* to a copyist, together with a reliability value: thus, the output of this step is represented by the pair of vectors $d=\left(d_{1},\;\ldots ,\;d_{s_{P}}\right)$ and $a=\left(a_{1},\;\ldots ,\;a_{s_{P}}\right)$, where $d_{i}$ is the label of the scribe assigned to the *i*-th sample in *P*, and $a_{i}$ the relative confidence degree. As mentioned in the introduction, the final classification step, which assigns the whole page to a single copyist, is performed by using the combining rule presented in Section 5.

## 4. Deep Learning for Writer Identification

**Figure 2:**
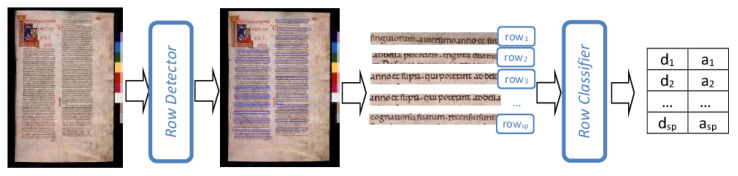
The deep learning (DL) System.

Deep Neural Networks represent a well-established category of artificial neural networks where the number of layers has become increasingly large. In the DNN realm, Convolutional Neural Networks (CNNs) have been proven as one of the most effective solutions able to work directly on raw data (images) and obtain an output that can be a “simple” score for image classification or a set of detected objects into a scene. The proposed DL system is based on two CNNs: the first one is intended to detect the text lines in each page of the manuscript, and the second one to assign each row to a single writer. An overview of the DL system architecture is shown in Figure 2.

The first module consists in a DL-based object detector aimed at automatically identifying the rows in each page of the manuscript. Several CNN architectures have been conceived for the detection of a set of objects of interest in an image. One of the most effective and scalable CNN for object detection is the MobileNetV2 [23], a very versatile network that can be suitably adapted also on devices with limited computing resources. To generate the detection map, we applied to the MobileNetV2 a Single Shot Detector (SSD) [24], namely the SSDLite object detector [23]. The main characteristic of MobileNetV2 and SSDLite is to use depth-wise separable convolutions that consists in applying two 1D convolutions with two kernels instead of performing a 2D convolution with a single kernel. In this way, fewer parameters and less memory are required for training, and the resulting model is very small and efficient.

The second module of the DL system is meant to classify the text lines identified by the row detector. For this purpose, we applied a CNN where we separately defined (i) a convolutional feature extractor used to obtain high-level features from the input images, and (ii) a meta-classifier for the final writer identification. The goal here is to decouple the feature extractor from the following classification step because the choice of the feature extractor affects processing time and performance of the detector. The architecture of the meta-classifier (see Table 1 for more details) consists of two fully connected layers with 2048 nodes separated with a dropout layer to improve the generalization capability of the network, and is terminated with a Softmax layer that generates a confidence degree in the range [0,1] for each class.

Both the CNNs for row detection and row classification have been trained through the transfer learning technique. In particular, the row classification model has been trained in two steps. In the first, a network model is retrained by tuning the parameters in just the final classification layers rather than all the weights within the feature extractor layers (here defined Transfer Learning—TL phase). This means that all the parameters of the feature extractor are frozen with values taken from a pre-trained model, whereas the parameters of the meta-classifier are randomly initialized and trained. On the contrary, in the second step, all the parameters of both feature extractor and classifier are unfrozen and retrained (here defined Fine-Tuning—FT phase). The proposed FT assumes that the parameters of the feature extractor are initialized with the values of the pre-trained model whereas the parameters of the meta-classifier are taken from the final model of the previous TL step.

Similarly to the case of LF system, let us denote with $s_{P}$ the number of rows automatically identified in the page *P* by the DL-based object detector: the output of the DL system is represented by the pair of vectors $d=\left(d_{1},\;\ldots ,\;d_{s_{P}}\right)$ and $a=\left(a_{1},\;\ldots ,\;a_{s_{P}}\right)$, where $d_{i}$ is the label of the scribe assigned to the *i*-th row in *P*, and $a_{i}$ the relative confidence degree. Also in this case, the final classification step, which assigns the whole page to a single copyist, is performed by using the combining rule presented in Section 5.

## 5. The Final Page Classification Step

As previously mentioned, the page classification step combines the responses provided by the writer identification system for each pattern detected in a manuscript page *P*, in order to reliably associate that page to a single scribe. Let us recall that for the LF writer identification system each pattern consists of *M* consecutive rows, while the DL writer identification system considers patterns composed of a single row. For both systems, we have denoted with $n_{W}$ the number of different copyists to be distinguished, with $s_{P}$ the number of patterns in a page *P* and with $d=\left(d_{1},\;\ldots ,\;d_{s_{P}}\right)$ and $a=\left(a_{1},\;\ldots ,\;a_{s_{P}}\right)$ the output vectors for that page, where the *i*-th element $d_{i}$ is the label of the scribe assigned to the the *i*-th pattern in *P*, and $a_{i}$ the relative confidence degree. We adopted a weighted majority vote rule as combining strategy, assuming the response on each pattern in *P* as a vote, weighted by the corresponding confidence degree. Thus, the page *P* is assigned to the writer $\hat{w}$ given by:

(1) $$\hat{w}=\arg max_{j=1,\;\ldots ,\;n_{W}}\sum_{i=1}^{s_{p}}a_{i}I\left(d_{i}=w_{j}\right)$$

where I(·) is an indicator function equal to 1 if $d_{i}=w_{j}$ and 0 otherwise.

**Figure 3:**
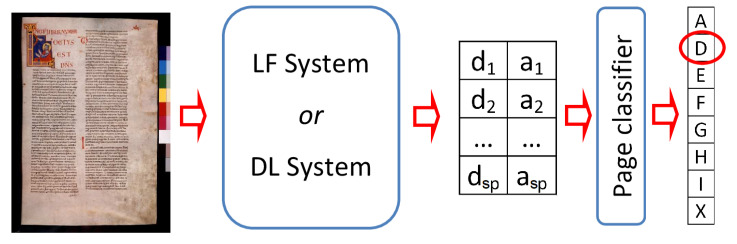
The architecture of the two end-to-end systems considered.

Therefore, a page *P* is classified as written by the copyist $w_{j}$ if most of its patterns (rows for DL system or groups of rows for LF system) are reliably identified as belonging to $w_{j}$. Figure 3 shows the whole end-to-end system.

## 6. Experimental Comparisons

In order to compare the performances of the proposed systems for page classification, we performed two sets of experiments. In the first one, we compared the classification performance of both systems in terms of accuracy. In the second one, we investigated the system behavior when a reject option is implemented. In the following subsections we first introduce the Avila dataset, then we report the comparison results for both the experiments.

### 6.1. The Avila Dataset

We compared the LF and DL systems on a large dataset of high-quality digital images obtained from a giant Latin copy of the whole Bible, known as the “Avila Bible”. It consists of 870 two-column pages handwritten in Italy within the third decade of the XII century. The pages written by each copyist are not equally numerous, the palaeographic analysis individuated 12 scribal hands that wrote from 1 to 143 pages. In several case, about 2% of the dataset, parts of the same page are written by different copyists.

In this work, to have sufficient data to be used in a deep learning approach, we considered 749 pages where eight writers can be identified. Each page was digitized with a resolution of 6000×4000 pixels and labeled by an expert paleographer as $\left\{w_{1},\;\ldots ,\;w_{n_{W}}\right\}$. Hereafter, by using the same labels chosen by the paleographers for characterizing two different periods and origins of copyists, we identified the copyists with the letters A, D, E, F, G, H, I, X. In particular, the first letters correspond to copyists that collaborated in handwriting in Italy within the third decade of the 12th century, while the scribe *X* belongs to the more recent the Spanish group, that completed the Bible text and decoration. It is worth noting that, in consideration of the choice done on the pages to be processed, if a page was written by different copyists, each page was labeled indicating the “prevailing” writer (i.e., the writer who wrote the greater part of the page). For this reason, the two systems will provide only one class for each page according to Equation (1), with the only difference on the meaning of $S_{p}$, that is the number of groups of *M* consecutive row in a page for LF system, while it represents the number of detected rows in the DL system.

### 6.2. Comparing the Performances of the Page Classification Systems

The two systems were trained and tested on 749 pages of Avila Bible. In particular, for the DL system we used all the pages because it needs the training of the row detector with a supervised procedure, whereas for the LF system we used only respectively 56 and 653 pages for training and testing the page classifier.

In the LF system the training and test sets were of 1117 and 14,340 feature vectors fv, respectively. They correspond to the groups of four consecutive rows analyzed in the page (in the following we will denote them as “fragments”). Note that each page may have from 4 to 13 fv per column. We used three effective and widely-used classification schemes to label the fragments extracted from the page images, namely Decision Tree (DT) [25], Random Forest (RF) [26] and Multilayer Perceptron (MLP) [27]. We used the implementation provided by the Weka tool, a popular collection of machine learning algorithms. As for the parameters for DT, RF and MLP we have used the default parameters provided by Weka.

In the DL system 12,099 rows were manually extracted from 96 pages (12 per writer). Such pages were then used to train both the row detector and the row classifier. The training and validation sets contained 7197 and 1871 rows corresponding to the rows extracted from seven and two pages per writer, respectively. To test the performance of the DL system, the remaining labeled 3031 images of rows, corresponding to the rows extracted from 24 pages (three per writer) were used for testing the whole DL system. These preliminary results are reported in [28]. Note that in this paper the remaining 653 pages were used to test the DL system and comparing its performance with the LF system. As for the row detector of the DL system, we used MobileNetV2 plus the SSDLite detector with the transfer learning technique. The model was previously trained on MS-COCO [29], a public well-known dataset, and then, it was retrained on the dataset of pages extracted from the Avila Bible. In particular, the MobileNetV2 usually works with a resized version of the input images, this for reducing the memory footprint, the training/inference time of the network, and at the same time to preserve a good sensibility in the detection of the rows. Further details about the choice of the optimal internal resolution for MobileNetV2 can be found in [28]. For the retraining, we used a root mean square propagation (RMSprop) with the following parameters: learning rate =0.004, batch size =24, and momentum =0.9. The training was stopped after 200,000 epochs with a patience of 200 epochs, i.e., it was stopped early if the validation loss did not improve for 200 epochs. As feature extractor in the row classifier model, we used five well-known DNN architectures, namely InceptionResNetV2 [30], InceptionV3 [31], NASNetLarge [32], ResNet50 [33] and VGG19 [34]. We considered these networks since they reached state-of-the-art performances in many computer vision applications, by introducing new structural elements (inception, residual, dropout, etc.). The five models were trained in fine-tuning and all the models were pre-trained on the ImageNet dataset [35]. Data augmentation has been also applied to the dataset, following the procedure detailed in [28]. Note that in our previous work [28], we also trained these networks from scratch, but the fine-tuning technique achieved a better performance. Table 2 shows the details of the feature extractors: input size, size $s_{bn}$ of the bottleneck, and number of parameters $N_{par}$. Using the meta-classifier architecture described in Section 4, the number of parameters $N_{par}$ to be trained in the meta-classifier depends on the size $s_{bn}$ of the bottleneck of the feature extractor, i.e., on the number of features extracted, and on the number of writers to be identified $n_{W}$ that in our case is equal to 8; we have:

(2) $$N_{par}=s_{bn}*2048+2048*2048+2048*n_{w}$$

The classification performance of both the architectures were evaluated, firstly, in terms of per-class and global accuracy, and then using the F1 score. The per-class accuracy $Acc_{i}$ for the *i*-th class was calculated as the number of samples correctly classified as class *i* divided by the number $n_{i}$ of samples belonging to the class *i*. The global accuracy, was computed as the number of correctly classified samples divided by the total number of samples $n=\sum_{i=1}^{n_{W}}n_{i}$. As concerns the F1 score, it is typically used for two-class problems and it is defined as the weighted harmonic mean of precision and recall. Because in our work we present a multi-class problem we can define the per-class recall $Rec_{i}$ equal to the per-class accuracy previously defined, whereas the per-class precision $Prec_{i}$ is evaluated as the number of samples correctly classified as class *i* divided by the number of samples classified as class *i*. The per-class F1 score is then defined as:

(3) $$F1_{i}=2\frac{Prec_{i}Rec_{i}}{Prec_{i}+Rec_{i}},$$

whereas we define a global F1 score as the weighted average of per-class F1 score when considering the class distributions:

(4) $$F1=\frac{1}{n}\sum_{i=1}^{n_{W}}n_{i}F1_{i}$$

The results in terms of the considered performance measures are shown in Table 3, where the last row reports the number of pages tested for each writer.

The best results for the LF system were achieved by the RF classifier. Indeed, 86.04% of global accuracy and 84.40% of F1 score outperform the second best results of DT classifier of 3.06 and 2.41 points in percentage, respectively, while, the MLP classifier achieved the worst performance.

For the DL system the best results were achieved by InceptionResNetV2 with 96.48% and of 96.56% of global accuracy and F1 score, respectively. While VGG19 and ResNet50 showed the lowest performance, with the 82.85% of ResNet50 accuracy and 82.74% of VGG19 F1 score. Such results confirm the behaviour shown by the DL system when it is used for row classification [28] but, in the case of the LF system, the application of the majority vote decision rule 1 to the page showed poor performance respect to previously published results [5]. Most probably, this worse result is due to that fact that we used a smaller training set. Indeed, in a previously published paper [36] a study on the minimum training data size was carried out considering the whole database. Note that the results achieved here follow those presented in [36], when the number of samples used for training was the 5% of the whole database. Indeed, the results presented for the LF system are related to a training set size of 7%. Therefore, the LF system evaluated on page recognition has confirmed a good performance even considering the higher computational complexity of the deep learning procedure.

### 6.3. Testing the Reject Option

In many applications, wrong predictions may cause losses much greater than those due to a withdrawn decision, i.e., the classifier does not label the sample, which is sent to a different automatic system or a human expert. In such a case, we say that the sample is “rejected”. The reject option can be implemented when the classification systems used, besides the predicted class, provides also the probability that the sample belongs to the class. Once this probability is available, the reject option can be implemented by rejecting the samples whose probability is below a given threshold. This last value depends on the application and it is typically set after drawing the so-called “error-reject” curve. This curve shows the relationship between the error and reject rates when the threshold value is varied, and allow the user to choose the threshold value that provides the couple of error-reject values that is best suited for the constraints and requirements of the application at hand. Typically, good curves are those where the error rate rapidly decreases while the reject rate does not increase too much. Chow [37] has proved that when the probability of the predicted class is well-estimated, this option allows any classification system to strongly reduce the error rate for the accepted samples, at the cost of a low reject rate.

In our case, the reject options implemented for the DL and LF systems are based on the page classification approaches detailed above: a page *p* is assigned to the writer whose number of rows (four-row fragments for LF) is the largest one in *p*. More specifically, if $n_{p}$ rows (four-row fragments for LF) have been detected and classified in *p*, then the probability that the page *p* belongs to the i-th writer is computed as the ratio $n_{i}/n_{p}$, where $n_{i}$ is the number of rows written by the i-th, according to the classifier output. Then the proposed system provides page labels only when they are deemed reliable, according to the just-mentioned rejection rule, whereas the remaining pages are rejected. The aim is to implement a semi-automatic system able to label the pages with a very low error rate, at the cost of demanding the decision to a human-expert for a reasonable number of pages.

**Figure 4:**
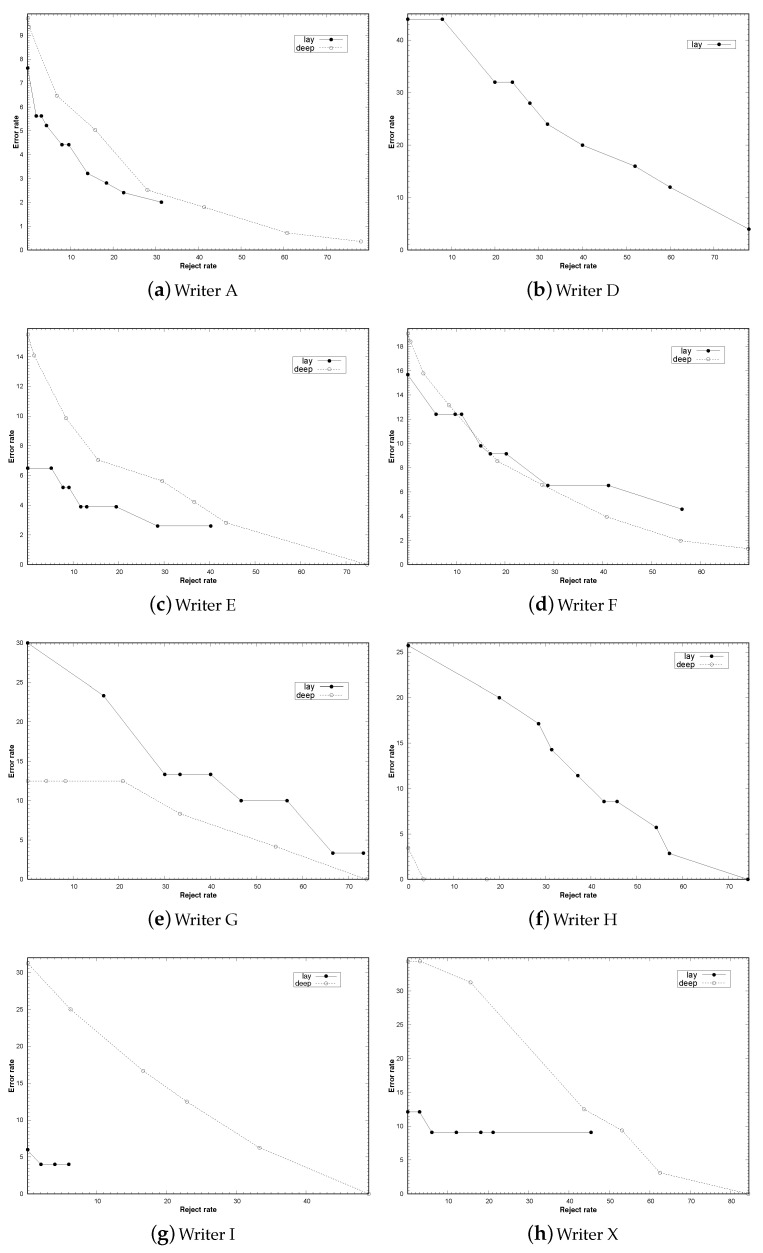
Error-reject curves for the different writers for the LF (lay) and DL (deep) system. Note that the rates are expressed in percentage.

To compare the performance of the reject options of the two systems, for each writer, we have plotted the error-reject curves (see Figure 4). These curves have been drawn using the results of the RF classifier and InceptionResNetV2 for the LF and DL system, respectively. Note that for the DL system the plot for the writer D is not shown because all his pages were correctly classified, even without setting any reject threshold.

As for the LF system, from Figure 4, we can see that the “starting point”, i.e., the error rate without reject option, represented by the most left point in the plot (we will refer to it in the following as $e_{0}$), differed widely among the different writers, ranging from about 44% of the writer D to about 6% of the writers H and I. In addition, the trends of the curves differed among them, with some of them showing a rapidly decreasing error rate, whereas for others this rate remained constant, after an initial decrease. The curves of the writers D and H belong to the first case, where a very low error was achieved at the cost of a very high rejection rate. Therefore, only very high output probability values allowed the LF system to classify reliably the samples of these writers. The curves for the writers I and X belonged to the second category and indicated that there was gap in the probabilities provided: part of the pages were classified with a low value, whereas the remaining ones were classified with a very high value. Moreover, it is worth noting that for the writer I, we achieved an error rate that can be considered acceptable (4%), at the cost of a very low reject rate (2%). As concerns the writer X, although the reject rate was low, the error rate decreased to about 9%.

As for the remaining writers, namely A, E, F, and G, their trends can be considered as intermediate cases between the two mentioned above. Indeed, in this case the error rate reduction of the whole curve was not so sharp like the case of writers D and H, but neither constant as for writers I and X. Therefore in this case, there was a significant number of misclassified samples with a low probability value. This implies that the first part of these curves was well-shaped, i.e., the error rate rapidly decreased while the reject rate did not increase too much. On the other hand, the same did not hold for the remaining samples, i.e., those characterized higher probability values. In this case, indeed, error reduction was much less sharp, indicating that many of these samples were correctly classified, even if the associated probability value was not so high.

Regarding the DL system, from Figure 4, we can see that also in this case $e_{0}$ differed widely among the different writers, ranging from about 34% of the writer X to 3.5% of writer H. In addition, the trend of these curves differed among them. For instance, for the writer A $e_{0}$ was about 10%, while accuracy close to 2% could be achieved by rejecting less than 30% of the test pages. Regarding the writer H $e_{0}$ was about 3.5% and the rejection of less than 4% of the pages allowed us to classify correctly all the test pages (0% of error rate). Finally, as for the writers I and X, $e_{0}$ was about 30% and the error rate decreased slowly, while error rates around the 10% were obtained only rejecting at least about 25% of the samples (writer I), or even about the 50% (writer X). From these results it is clear that the performance of the proposed reject option depended on that of the classifier, thus providing satisfactory results only when the latter achieved accuracy at least around 10%.

These results confirm that for both systems the effectiveness of the probability estimation achieved by the reject option implemented depended on the starting point, or even better by the performance, obtained on the single writer, of the classification system used. It is also worth noting that the curves of both systems exhibited similar trends. Most probably this depended on the fact that both systems used the same reject rule, as well as the same page classification system, based on the predictions of the rows (DL) or of the fragments (LF). However, comparing the curve trends of the two systems, those of the DL system were better shaped. Indeed, in most cases their error rates decreased more sharply than the corresponding LF curve. This confirms that the DL system provided a better estimation of the classification probabilities.

## 7. Conclusions

Palaeography aims at studying mediaeval handwritings and the availability of effective image analysis algorithms, as well as that of high-quality digital images gave rise to the development of new applications for the automatic processing of ancient manuscripts. In previous research activities in this framework, we developed two end-to-end systems able to assign each page image of a Latin book given to one of the writers who contributed to the writing process of such a book. The first system uses a set of features to characterize highly standardized handwriting and book typologies according to the suggestions of the paleographers, whereas the second one makes use of deep learning techniques, based on transfer learning.

In this study we have tried to answer the following question: do DL-based approaches represent a general methodology for automatically designing machine learning systems for palaeography applications? To this aim we compared the performance of a DL based approach with that of a “classical” machine learning one on a large dataset of images extracted from an entire 12th-century Bibles, the “Avila Bible”, where some basic features, directly derived from page layout analysis, can be easily extracted by using standard image processing algorithms and allow the development of effective machine learning systems for scribe identification.

The systems have been compared in two different ways. First we compared the accuracies achieved on each writer, then we tested the ability of both systems in implementing an effective reject option. In both experiments the DL-based system, without using any domain knowledge, has proved to be able to achieve results comparable to or even better than those obtained by a classical machine learning approach, which need the knowledge provided by expert paleographers.

## Figures and Tables

**Table 1:** Architecture of the meta-classifier.

Layer	Type	Input Size	Output Size	Kernel Size	Rate
1	Fully connected	$s_{bn}$	2048	1×1	
2	Dropout	2048	2048		0.5
3	Fully connected	2048	$n_{W}$	1×1	
4	Softmax	$n_{W}$	$n_{W}$		

**Table 2:** Details of the feature extractors.

Model	Input Size	$s_{\mathrm{bn}}$	$N_{\mathrm{par}}$
VGG19	256×256	512	5,259,264
ResNet50	224×224	2048	8,404,992
InceptionV3	299×299	2048	8,404,992
InceptionResNetV2	299×299	1536	7,356,416
NASNetLarge	331×331	4032	12,468,224

**Table 3:** Per-class and global performance obtained in page writer identification task in LF and DL systems.

Model	Measure	Writers								Global Performance
		A	D	E	F	G	H	I	X	
LF-DT	Acc	0.8811	0.9765	0.9695	0.8987	0.9730	0.9678	0.9872	0.9296	0.8298
	F1	0.8549	0.7111	0.8903	0.8026	0.7541	0.7273	0.9278	0.5495	0.8199
LF-RF	Acc	0.9237	0.5200	0.9377	0.8366	0.7000	0.7429	0.9400	0.8788	0.8604
	F1	0.8647	0.6667	0.9211	0.8421	0.7200	0.8254	0.9592	0.6105	0.8440
LF-MLP	Acc	0.7867	0.9505	0.9408	0.7880	0.9427	0.9486	0.9809	0.9165	0.7071
	F1	0.7573	0.1429	0.8079	0.6331	0.3913	0.5455	0.9143	0.5333	0.6830
DL-VGG19	Acc	0.9281	0.9474	0.5915	0.8947	0.7500	0.9655	0.6875	0.3125	0.8315
	F1	0.9264	0.6667	0.6412	0.8635	0.6316	0.9655	0.7674	0.4167	0.8274
DL-ResNet50	Acc	0.9065	1.0000	0.7324	0.7961	0.7083	0.8966	0.5000	0.9375	0.8285
	F1	0.9097	0.6230	0.7879	0.8203	0.5965	0.9286	0.6154	0.8219	0.8307
DL-InceptionV3	Acc	0.9065	1.0000	0.8732	0.8289	0.9167	0.9655	0.9167	0.9688	0.8943
	F1	0.9351	0.8837	0.9254	0.8571	0.7458	0.9655	0.8544	0.8158	0.8970
DL-InceptionResNetV2	Acc	0.9928	1.0000	0.9859	0.9145	0.9583	0.9655	0.9167	0.9688	0.9648
	F1	0.9910	0.9048	0.9859	0.9521	0.9388	0.9655	0.9462	0.8493	0.9656
DL-NASNetLarge	Acc	0.9784	1.0000	0.8873	0.8816	0.9583	0.9655	0.8750	0.9688	0.9372
	F1	0.9680	0.9500	0.9265	0.9241	0.8364	0.9655	0.9130	0.8493	0.9379
Number of pages per class		278	19	71	152	24	29	48	32	653
